# Nanogenerators for Self-Powered Gas Sensing

**DOI:** 10.1007/s40820-017-0146-4

**Published:** 2017-05-06

**Authors:** Zhen Wen, Qingqing Shen, Xuhui Sun

**Affiliations:** Institute of Functional Nano and Soft Materials (FUNSOM), Jiangsu Key Laboratory for Carbon-Based Functional Materials and Devices, and Collaborative Innovation Center of Suzhou Nano Science and Technology, Soochow University, Suzhou, 215123 People’s Republic of China

**Keywords:** Nanogenerator, Self-powered, Gas sensing, Piezoelectric, Triboelectric

## Abstract

Looking toward world technology trends over the next few decades, self-powered sensing networks are a key field of technological and economic driver for global industries. Since 2006, Zhong Lin Wang’s group has proposed a novel concept of nanogenerators (NGs), including piezoelectric nanogenerator and triboelectric nanogenerator, which could convert a mechanical trigger into an electric output. Considering motion ubiquitously exists in the surrounding environment and for any most common materials used every day, NGs could be inherently served as an energy source for our daily increasing requirements or as one of self-powered environmental sensors. In this regard, by coupling the piezoelectric or triboelectric properties with semiconducting gas sensing characterization, a new research field of self-powered gas sensing has been proposed. Recent works have shown promising concept to realize NG-based self-powered gas sensors that are capable of detecting gas environment without the need of external power sources to activate the gas sensors or to actively generate a readout signal. Compared with conventional sensors, these self-powered gas sensors keep the approximate performance. Meanwhile, these sensors drastically reduce power consumption and additionally reduce the required space for integration, which are significantly suitable for the wearable devices. This paper gives a brief summary about the establishment and latest progress in the fundamental principle, updated progress and potential applications of NG-based self-powered gas sensing system. The development trend in this field is envisaged, and the basic configurations are also introduced.

## Introduction

With the rapid development of internet of things (IoTs) and machine to machine (M2M) technology, the requirement of wireless, sustainable, multi-functional and independent operation of sensing networks has been becoming increasingly important [1–4]. Considering the large number and small scale of sensors, the implantation of traditional power supply will be a big challenge, and developing self-powered sensors that can employ the ambient environmental energy and not dependent on a battery or external power source is highly desired [5–7]. These innovative self-powered sensors have been fabricated profiting from the recent advances in environmental energy harvesting technologies, which open an access for developing environmental friendly, independent, remote and mobile, maintenance-free operating nanodevices. The concept of self-powered sensors is based on coupling an external energy harvesting or powering unit, such as a solar cell or electromagnet generator, with the functional nanodevice of concern to fulfill the demand for the operation [8, 9].

However, the intermittent and unpredictable nature of solar energy as well as the complexity and heavy weight of electromagnet generator is an inevitable challenge for their expansion as a reliable power supply system to some extent. Since 2006, Zhong Lin Wang’s group has firstly fabricated a novel piezoelectric nanogenerator (PENG) that could convert environmental mechanical energy into electrical energy by means of piezoelectric zinc oxide nanowires (NWs) [10]. After that, in early 2012, they invented a triboelectric nanogenerator (TENG) based on the conjunction of triboelectrification and electrostatic induction with higher output and more available materials [11–13]. Most recently, Wang has presented the fundamental theory of the NGs starting from the Maxwell equations. In the Maxwell’s displacement current, the second term ∂P/∂t in the Maxwell’s displacement current is directly related to the output electric current of the nanogenerators (NGs), meaning that NGs are the applications of Maxwell’s displacement current in energy and sensors [14].

As new power generation technologies, NGs can be used to convert mechanical energy into electricity; on the other hand, by analyzing the electrical output signals (including *V*_oc_, *J*_sc_, frequency, etc.), information on the mechanical input (magnitude and frequency) can be successfully retrieved [15–19]. Since this sensing technology originates from the output signals of the NG itself, no external power source is required to apply onto the device, which is a unique advantage over conventional sensing technologies. Since then, by correlating the mechanical input with many other parameters, various self-powered prototypes have been realized for different practical applications, mainly including two aspects, one is physical sensing system, such as pressure detection [20, 21], motion sensing [22, 23], acoustic sensing [24, 25], security check [26, 27], medical science [28, 29], and even implantable biosensors [30, 31], and another is chemical sensing system, such as water splitting [32, 33], cleaning pollution [34, 35], anti-corrosion protection [36, 37], electrochromic reaction [38, 39], and electrochemical active sensor [40, 41]. Among above-mentioned applications, monitoring the concentration of gases is one of the most important permanent requirements in many fields of industrial processes as well as in everyday life. Applications typically involve safety supervision of important process parameters, environmental monitoring and issues concerning the air quality [42–45]. Owing to the high demand for reliable, independent, minimization, sustainable, maintenance-free and continuous operation of gas sensing devices, one major field is the improvement in self-powered system. The self-powered gas sensing system based on NGs will be an optimum solution.

In reviewing the studies, the recent works have shown promising concept to realize self-powered gas sensors that are capable of detecting gases using NGs as external power sources to actively generate a readout signal or to activate the sensor–gas interaction. These self-powered gas sensors drastically reduce power consumption compared with conventional sensors and additionally reduce the required space for integration. Herein, the objective of this paper focuses on a review of the fundamental principle, updated progress and potential applications of NG-based self-powered gas sensing system. The development trend in this field is envisaged, and the basic configurations are also introduced.

## PENG-Based Self-powered Gas Sensing System

**Fig. 1:**
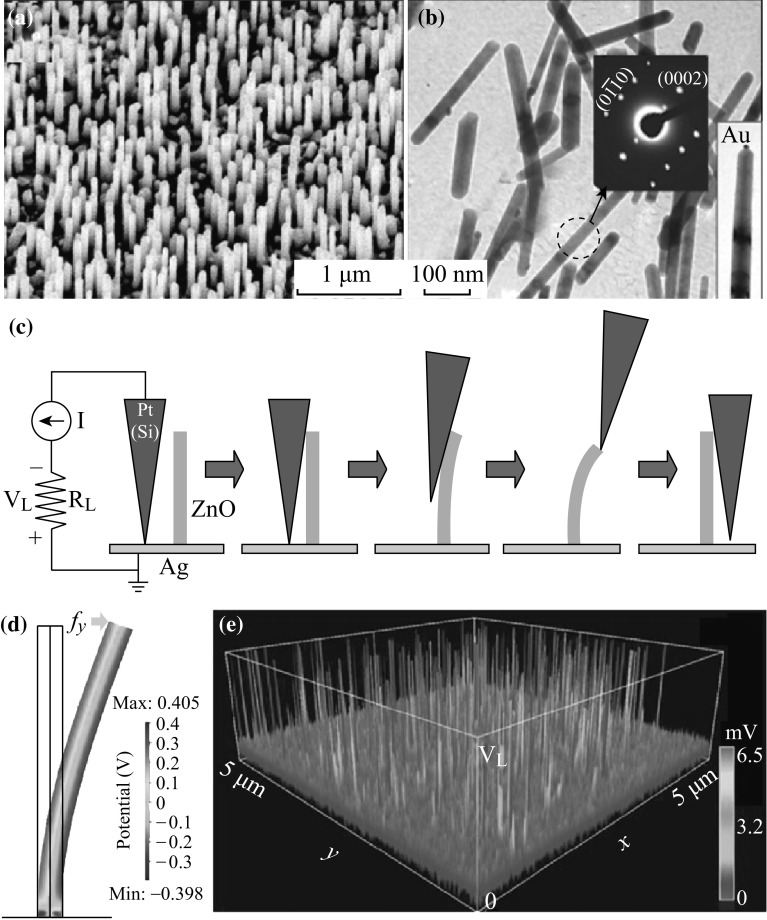
Design for converting nanoscale mechanical energy into electrical energy by a vertical piezoelectric ZnO NW-based PENG. **a** SEM images of aligned ZnO NWs grown on *α*-Al_2_O_3_ substrate. **b** TEM images of ZnO NWs, showing the typical structure of the NW without an Au particle or with a small Au particle at the top (*inset* at center shows SAED pattern). **c** Experimental setup and procedures for generating electricity by deforming a piezoelectric NW with a conductive AFM tip. **d** Potential distribution for a ZnO NW in *side view*. **e** Electric signal collected by AFM on a ZnO NW array. Figures adapted from [10, 46]

The concept of a PENG was firstly presented for converting nanoscale mechanical energy into electricity in 2006, by scanning across vertical piezoelectric ZnO NW with a conductive atomic force microscope (AFM) tip, as shown in Fig. 1 [10]. The fundamental of electricity generation in piezoelectric materials is the breaking of central symmetry in the crystal structure under external force, thereby forming a piezoelectric potential, or piezopotential. When a piezoelectric NW is deflected, a piezoelectric potential can be generated on the side surfaces due to the crystal lattice distortion. For ZnO NWs, the tensile side surface gives a positive potential, while a negative potential appears on the compressive side surface [47, 48]. In 2012, Seong Min Kim and co-workers theoretically investigated that external surface charges on ZnO NWs or AlN nanotubes (NTs) can affect the piezoelectric behavior under uniform compression. The free-carrier depletion caused by negative surface charges via surface functionalization on vertically compressed ZnO and AlN NWs/NTs indicates the enhancement of piezoelectric potential is due to the free carriers being fully depleted at the critical surface charge density [49, 50]. Since then, substantial progress has been made to couple the piezoelectric and functional properties, aiming at achieving PENG-based self-powered sensing system [6, 51]. On the basis of the piezoelectric-semiconductor materials such as ZnO, GaN and CdS with wurtzite or zinc-blende structure, the emerging field of PENG-based self-powered sensing system has been demonstrated in different forms, such as self-powered UV sensor [52] and self-powered pH sensor [53].

Recently, a new research field mainly aimed at constructing a portable gas sensing system without using external electric power. By coupling the piezoelectric and gas sensing characteristics, the piezoelectric output of PENG could act as both the power source and sensing signal. The gas adsorption on the surface of piezoelectric-semiconductor nanomaterials can change the free-carrier density, which can vary the piezoelectric output upon applied deformation through the piezoelectric screening effect. The key to realize the self-powered sensor is to choose the appropriate piezo-gas sensing materials. In this section, we would like to introduce the PENG-based self-powered gas sensing system systematically in the form of nanomaterials.

### Pure ZnO Nanomaterials

ZnO, benefiting from lacking central symmetry in wurtzite structures, exhibits strong piezoelectric properties in addition to having a large exciton binding energy and an excellent gas sensing property [54, 55]. Thus, it has potential for application in diverse fields, particularly in realizing a new generation of self-powered sensing system [56–58].

**Fig. 2:**
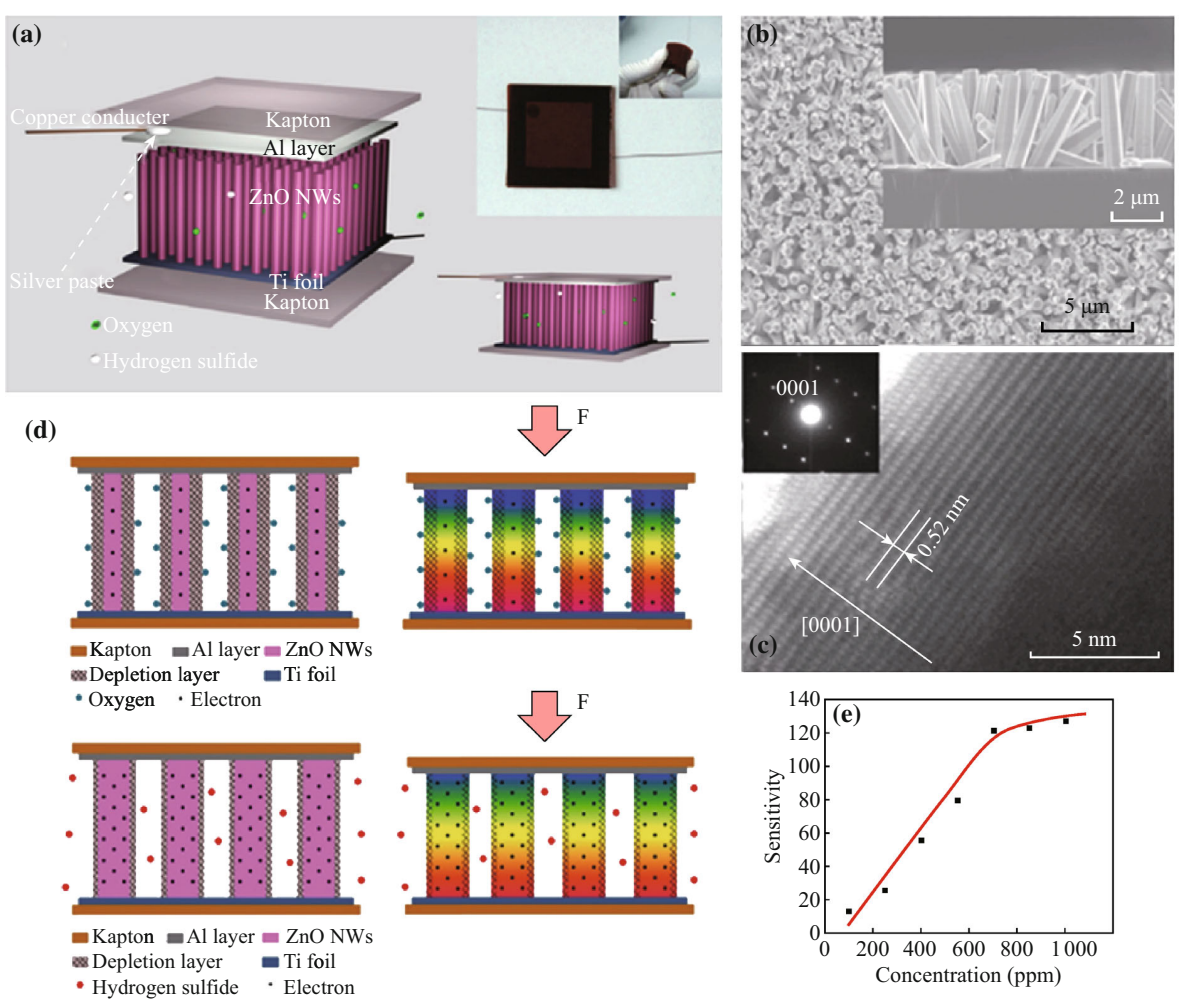
Surface free-carrier screening effect on the output of a ZnO NW-based PENG and its potential as a self-powered active gas sensor. **a** Schematic diagram showing the structural design of the PENG (the *inset* is a photograph of a typical device). **b** SEM image of ZnO NW arrays grown on Ti foil in a top view (the *inset* shows SEM the cross-sectional view image). **c** HRTEM and SAED pattern taken from the tip region of a ZnO NW. **d** The working mechanism of the PENG was driven by compressive strain. **e** The dependence of sensitivity on the ranging concentrations of H_2_S vapor. Figures adapted from [59]

In this regard, Xinyu Xue and co-workers, for the first time, reported the application of an unpackaged PENG based on ZnO NWs as a self-powered active H_2_S gas sensor, as shown in Fig. 2. This device is composed of three major components: Ti foil acts as both the substrate for the ZnO NW arrays and the conductive electrode that collects the piezoelectric voltage signal generated by ZnO NWs when being deformed by external compression. As the counter-electrode, a sheet of flexible Al foil is positioned on top of the ZnO NW arrays. In order to ensure good contact, the device was beat repeatedly for more than 40 min by a vibration shaker. Two sheets of Kapton board are fixed at two sides as bracing frames (Fig. 2a). Scanning electron microscope (SEM) images of top view and cross-sectional view of the ZnO NW arrays, respectively, reveal their diameters of 500 nm and lengths of ~5 μm (Fig. 2b). A high-resolution transmission electron microscope (HRTEM) image and the corresponding selected area electron diffraction (SAED) pattern taken from the tip region of a ZnO NW indicate that the ZnO NW is single crystalline with a length direction along the *c*-axis (Fig. 2c). As the *c*-axis of ZnO NW is under externally applied deformation, a piezoelectric field is created along the surface. The output of a PENG fabricated using ZnO NW arrays is largely influenced by the density of the surface charge carriers at the NW surfaces. Adsorption of gas molecules could modify the surface carrier density through a screening effect; thus, the output of the PENG is sensitive to the gas concentration (Fig. 2d). The demonstrated sensitivity to H_2_S to a level is as low as 100 ppm. The sensitivity against 100, 250, 400, 550, 700, 850 and 1000 ppm H_2_S is about 13.1, 25.5, 55.7, 79.3, 121.7, 122.8 and 127.3%, respectively (Fig. 2e). It is the first work which demonstrates the piezoelectric signal generated by pure ZnO NWs acts not only as a power source, but also as a response signal to the gas, suggesting a possible approach as a self-powered gas sensor [59]. The piezoelectric and gas sensing properties of ZnO NWs are coupled into one single physical process through the atmosphere-dependent screen effect on the piezoelectric output. This new mechanism opens a new direction for the development of the next generation of gas sensors and expands the scope of self-powered nanosystems.

Followed work of 2D ZnO nanosheets (NSs) network for a room-temperature self-powered humidity sensor has also been demonstrated [60]. However, compared with the traditional metal oxide semiconductor-based gas sensors, it is highly expected to achieve high response and selectivity of self-powered active gas sensor. Much efforts have been made to improve the sensing performance, including doping noble metal, introducing heterostructures, decorating proper element and UV enhancement [61–63].

### Noble Metal-Doped ZnO Nanomaterials

In gas sensing field, the conductivity response is determined by the efficiency of catalytic reactions of sensing materials with detected gas taken place at the surface of gas sensing materials. The control of catalytic activity of gas sensor material is one of the most commonly used methods to enhance the performance, especially in detection of low reducing gases, such as ethanol vapor, H_2_ and CO. Therefore, the pure ZnO thin film exhibits a very poor sensitivity and selectivity [64, 65]. Usually, noble metals are high-effective catalysts, which can be used to enhance the reactions on gas sensor surfaces. Thus, if the noble metal decoration can be introduced into self-powered active gas sensing, then higher-performance gas sensor can probably be realized.

**Fig. 3:**
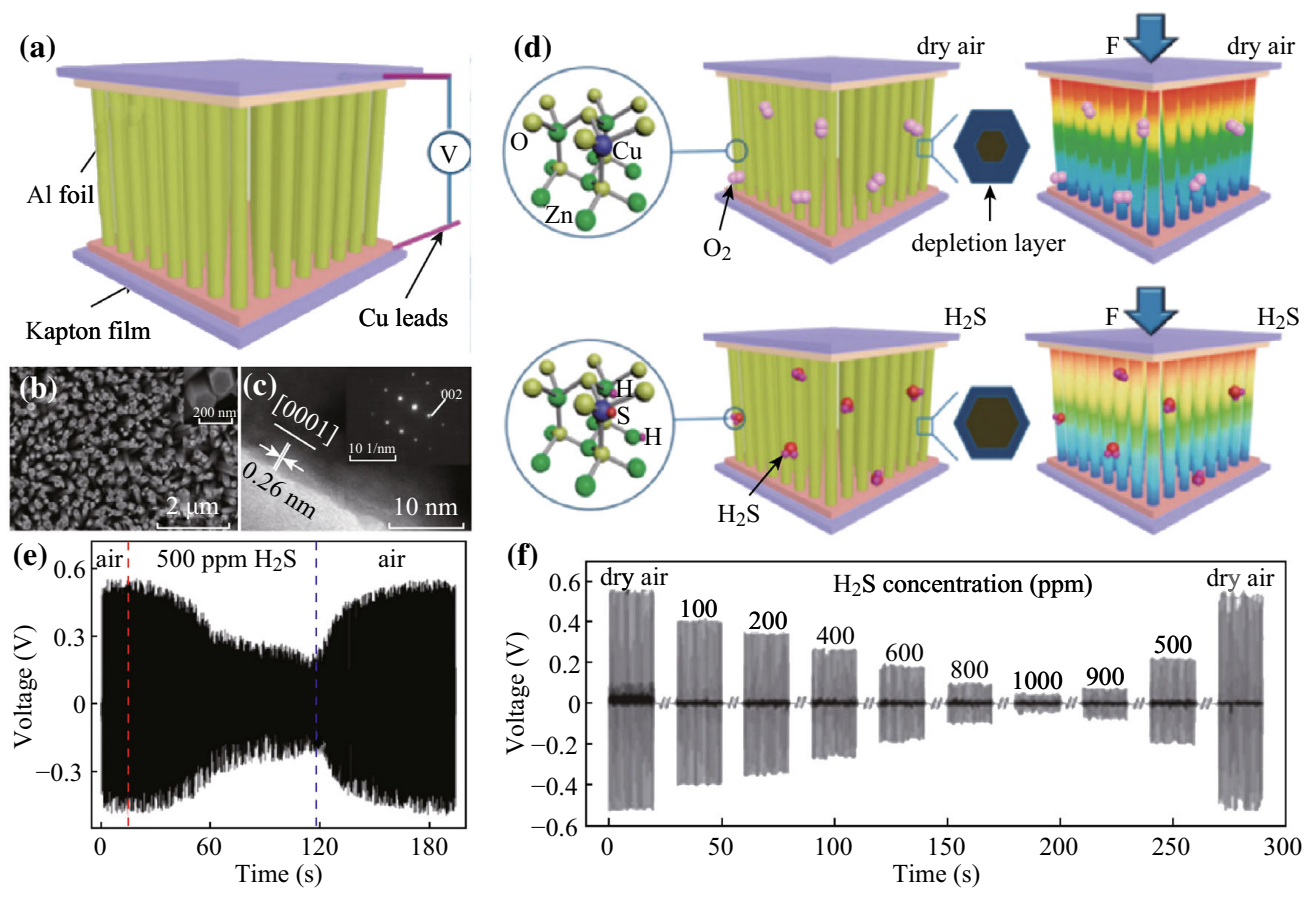
High response and selectivity of a Cu–ZnO NW PENG as a self-powered/active H_2_S sensor. **a** Schematic of a Cu–ZnO NW PENG as a self-powered/active H_2_S sensor. **b** SEM image of 5 at% Cu–ZnO NWs in top view. **c** HRTEM image and the SAED pattern of the edge region of a 5 at% Cu–ZnO NW. **d** Working mechanism of Cu–ZnO NW PENG as a self-powered/active H_2_S sensor. **e** A continuous responding-recovering process of the device against 500 ppm H_2_S. **f** Piezoelectric output voltage of the device under compressive force at room temperature in dry air and various concentrations of H_2_S. Figures adapted from [66]

In this case, Fu et al. realized a room-temperature self-powered H_2_S sensing with high response and selectivity from a Cu–ZnO NW-based PENG, as shown in Fig. 3 [66]. The brief fabrication of a Cu–ZnO-based self-powered/active H_2_S sensor was composed of three major parts: Cu–ZnO NWs as the power source and the sensing material, Ti and Al foils as the electrodes and Kapton films as the frames (Fig. 3a). The diameter and length of 5 at% Cu–ZnO NWs are ~230 nm and ~3.5 μm, respectively (Fig. 3b). A typical HRTEM image and corresponding SAED pattern show that the Cu–ZnO NW grows along the [0001] direction, and no secondary growth, visible defects or stacking fault can be observed (Fig. 3c). The enhanced room-temperature H_2_S sensing performance can be attributed to the coupling of the piezoelectric screening effect of ZnO NWs and the synergistic effect of the Cu dopant. Cu element has a similar ionic radius and electronic shell structure as Zn. Cu atoms can replace either substitutional or interstitial Zn atoms in the ZnO lattice, and Cu has higher attraction to H_2_S than Zn for the initial bridge-top (H-SH) configuration (Fig. 3d). A continuous responding-recovering process under compressive force (30 N, 1 Hz) at room temperature against 500 ppm H_2_S displays that the response and recovery time are 100 and 60 s, respectively (Fig. 3e). Upon exposure to 1000 ppm H_2_S, the piezoelectric output voltage of the device under compressive force decreases from 0.552 to 0.049 V, and the response is up to 1045, over 8 times larger than that of undoped ZnO (Fig. 3f).

Such a development of Cu–ZnO-based self-powered active H_2_S sensor is an important step for the practical applications in actively detecting gases at room temperature. The catalytic property of Cu greatly improved the sensing performance. This study demonstrates that introducing elemental doping into the self-powered active gas sensor is a very effective way to enhance its piezo-gas sensing performance. Meanwhile, this study could stimulate research into designing a new series of gas sensors for detecting more gas species at room temperature [66–70].

### ZnO-Based Heterostructures

Pure metal oxide materials appear favorable in some functional properties, but very few of them are suitable to all requirements. The heterostructures have been confirmed to improve the performance of metal oxides against various gases. More recent works have reported that upon exposure to the detected gas, the introduction of heterostructures significantly increases or decreases the resistance of the heterostructured nanomaterials, resulting in extremely high sensitivity. Also, the heterostructures can be introduced into self-powered active gas sensors to realize room-temperature gas sensor with high sensitivity.

**Fig. 4:**
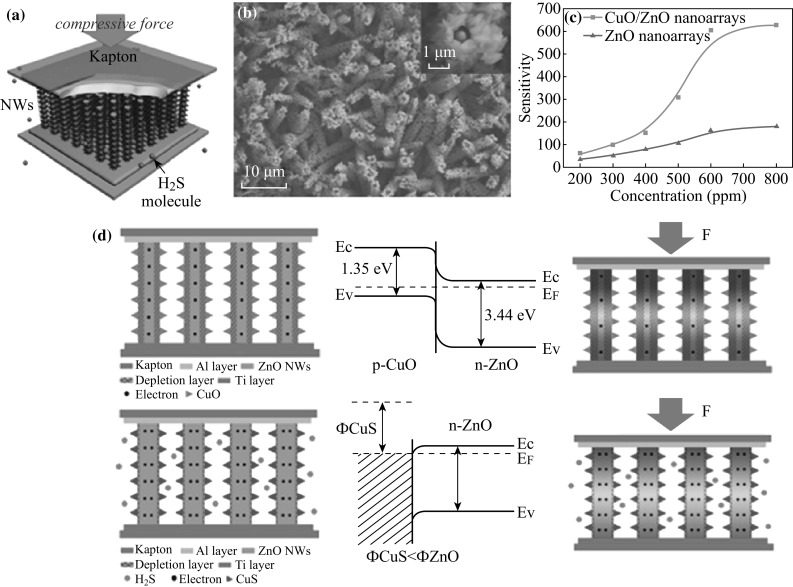
Conversion of PN junction influencing the piezoelectric output of a CuO/ZnO nanoarray PENG and its application as a room-temperature self-powered active H_2_S sensor. **a** Schematic diagram showing the structure of the self-powered active gas sensor with two Kapton boards as the frame. **b** SEM image of CuO/ZnO nanoarrays on the *top view* (the *inset* is an enlarged view of a selected area). **c** The sensitivity of the device upon exposure to different concentrations of H_2_S with one device repeatedly compressed by a constant applied strain of 0.012% at a frequency of 0.8 Hz. **d** The H_2_S sensing mechanism of the self-powered active gas sensor based on CuO/ZnO PN-junction nanoarrays driven by compressive strain. Figures adapted from [71]

Nie et al. have fabricated a CuO/ZnO heterostructure nanoarray-based self-powered/active gas sensor for room-temperature H_2_S detection, as shown in Fig. 4 [71]. The self-powered active gas sensor contains CuO/ZnO PN-junction nanoarrays as both piezoelectric and gas sensing materials. Flexible Kapton films as substrates can follow the height profiles of the NW arrays and make effective contacts between the tips of NWs and electrodes (Fig. 4a). The top-view SEM image of CuO/ZnO PN-junction nanoarrays indicates that CuO nanocones are uniformly distributed on the whole surface of ZnO NW arrays with the thickness of about 400 nm (Fig. 4b). Upon exposure to 800 ppm H_2_S at room temperature, the piezoelectric output of the device greatly decreases from 0.738 V (in air) to 0.101 V. The sensitivity increases to 629.8, much higher than that of bare ZnO nanoarrays (Fig. 4c). As the device was exposed to H_2_S, the CuO/ZnO PN junction was converted into a CuS/ZnO ohmic contact, which greatly increased the electron density in the NW and enhanced the screen effect on the piezoelectric output (Fig. 4d).

This result could stimulate a research trend on designing new composite piezoelectric material for high-performance self-powered active gas sensors. The combination of other components, such as organic composite, were also investigated. The high feasibility of organic surface modifications in terms of functional groups, as well as their steric and electronic structures might possibly enable the targeted design of various specific gas sensors.

**Fig. 5:**
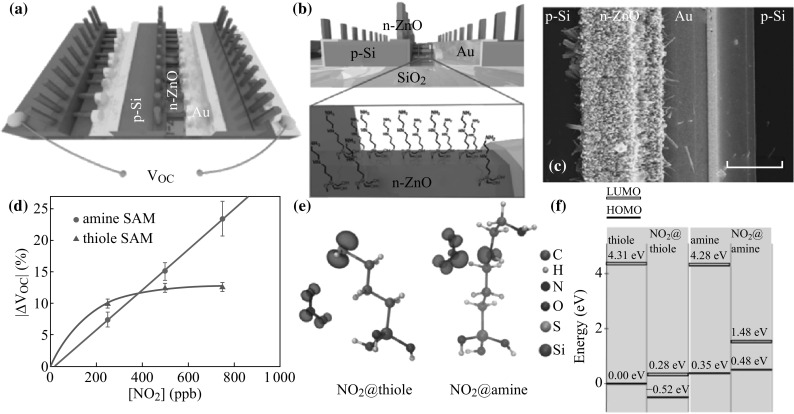
A highly selective and self-powered gas sensor via organic surface functionalization of *p*-Si/*n*-ZnO diodes. **a** Schematic of a singular p-n unit composed of *p*-Si *μ*-trenches. **b** The surface of the *n*-ZnO NWs was modified by adding a SAM of functional groups. **c** SEM image of a singular *p*-Si/*n*-ZnO diode (*scale bar*: 10 μm). **d** Diagram of the Δ*V*_oc_ response for different NO_2_ concentrations, for amine- and thiol-functionalized devices. **e** Most stable geometries of NO_2_ molecules adsorbed at the thiol and amine functionalities. **f** Energy levels of the HOMO and LUMO of the functionalities without adsorbed NO_2_ and with adsorbed NO_2_. The averaged electrostatic potential from the core region of the Si atom of each functionality was used as reference potential. The HOMO level of the isolated thiol functionality was set to 0 for convenience. Figures adapted from [72]

Martin W. G. Hoffmann and co-workers have demonstrated a selective and self-powered gas sensor by microfabricated *p*-Si/*n*-ZnO diodes upon visible-light illumination, as shown in Fig. 5. The selective sensing qualities were introduced by the functionalization of the *n*-ZnO surface with amine- as well as thiol-terminated organic self-assembled monolayer (SAM), capable of detecting low NO_2_ concentrations in the ppb range without the need of an external power source (Fig. 5a, b). After patterning of a *p*-Si layer on SiO_2_ via reactive ion etching, photolithographic methods were used to deposit layers of 20 nm of ZnO selectively on the *p*-Si sidewalls that served as a seed layer for site selective growth of *n*-ZnO NWs, to form *p*-Si/*n*-ZnO heterojunctions (Fig. 5c). Furthermore, the use of an organic SAM facilitated the gas–surface interaction without the need of heat or UV activation, as is required for bare inorganic gas sensors (Fig. 5d). Detailed density functional theory (DFT) simulations of the SAM–NO_2_ binding interactions and subsequent changes of the organic surface group frontier molecular orbitals indicate that the nature of the chemical SAM structure directly determines the gas response of the hybrid material (Fig. 5e). The contrary relative changes of the ionization potential and electron affinity upon NO_2_ binding for amine- and thiol-terminated SAMs correlate well with the experimentally observed sensing results (Fig. 5f).

This work gives an insight into the complex sensing mechanism of inorganic–organic hybrid gas sensors and shows the feasibility of transferring chemical signals from specific organic–gas interactions into active electronic signals solely driven by visible light [72]. Heterostructures can stimulate a research trend on the development of the next generation of room-temperature gas sensors and will further expand the scope for self-powered nanosystems.

### Non-ZnO Nanomaterials

It should be noted that ZnO NWs in the prototype piezo-driven active gas sensors may be corroded by H_2_S. Therefore, there is an urgent demand to explore new nanostructured materials that have good gas sensing performance, high piezoelectric output and high chemical stability as well.

**Fig. 6:**
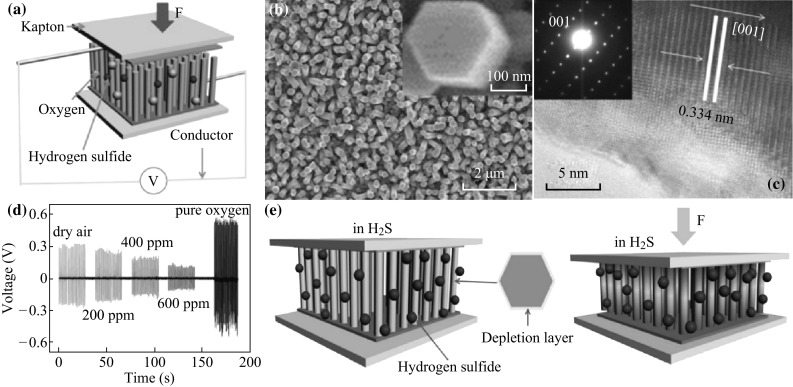
Synthesis of CdS nanorod arrays and their applications in flexible piezo-driven active H_2_S sensors. **a** Schematic diagram showing the structural design of the piezo-driven active H_2_S sensor based on CdS nanorod arrays. **b** SEM image of CdS NR arrays grown on Ti foil in a top view (the *inset* is an enlarged view of a selected area). **c** HRTEM and SAED pattern of the tip region of a CdS nanorod. **d** The piezoelectric voltage response of a device in dry air, H_2_S and pure oxygen at room temperature. The working mechanism of a CdS piezo-driven active H_2_S sensor. **e** The carrier density and depletion layer in CdS nanorods in H_2_S without compression and piezoelectric output under mechanical deformation. Figures adapted from [73]

By coupling the piezoelectric and gas sensing properties of CdS nanorods (NRs), a flexible piezo-driven self-powered/active H_2_S sensor has been fabricated by Penglei et al. [73], as shown in Fig. 6. The piezo-driven H_2_S sensor was fabricated from three parts: CdS NR arrays, Ti foil and Al layer as electrodes, and Kapton boards (Fig. 6a). The CdS nanorods are vertically aligned on the Ti substrate with the average diameter of about 200 nm. The inset of Fig. 6b shows that the cross-sectional shape of the CdS nanorods is hexagonal. The HRTEM image of the tip region of a CdS nanorod and the corresponding SAED pattern reveal that the CdS nanorod is single crystalline with a growth along the [001] direction (Fig. 6c). Upon exposure to 600 ppm H_2_S, the piezoelectric output of the device decreased from 0.32 V (in air) to 0.12 V. Such a flexible device can be driven by the tiny mechanical energy in our living environment, such as human finger pinching (Fig. 6d). When the device in H_2_S is under compressive strain, the screening effect of free electrons is extremely strong because of the high electron density, and the piezoelectric output is lower than that in air (Fig. 6e).

## TENG-Based Self-powered Gas Sensing System

**Fig. 7:**
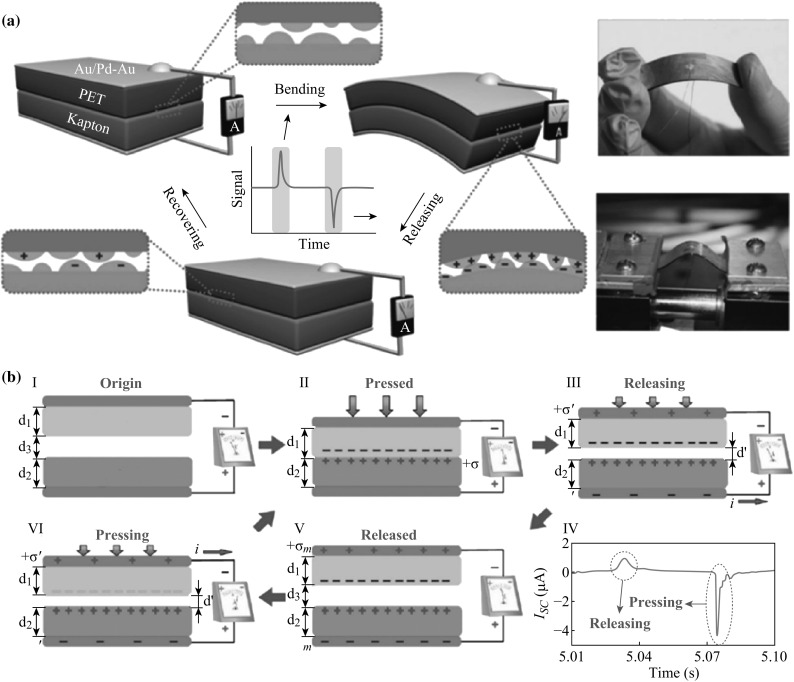
Invention and working principle of the first TENG that converts mechanical energy into electricity. **a** The construction and photography images in bending and releasing process and related electrical measurement tests. **b** Sketches that illustrate the operating principle. Figures adapted from [11, 74]

Triboelectrification has been conventionally known since the ancient Greek era and usually taken as a negative effect. However, tactfully based on a conjunction of triboelectrification and electrostatic induction, in 2012, a simple, cost-effective and all-polymer-based flexible TENG was invented by Zhong Lin Wang’s group, as shown in Fig. 7 [11, 75]. A TENG mainly consists of two polymer films that have different electron-attracting abilities, with metal films deposited on their back sides as electrodes (Fig. 7a). When the two films contact, friction happens, owing to the natural nanoscale surface roughness, which leads to equal amount but opposite signs of charges generated on the two films’ surfaces. Thus, an electric potential is formed at the interface region. When the two films contact and separate, the alternative potential will drive electrons in the external load to flow back and forth (Fig. 7b) [76–81]. Based on such a principle, four different modes of TENGs were invented, vertical contact-separation mode, lateral sliding mode, single-electrode mode and freestanding triboelectric-layer mode, respectively [82–87].

In the basic working principles of TENGs, the amplitudes of generated signals are all proportional to the triboelectric charge density when all of the other conditions are maintained the same [88–90]. The triboelectric charge density is greatly influenced by the surface alteration of certain chemical molecules or environmental factors, so self-powered electrochemical active sensors based on TENGs can be developed [91, 92]. In comparison with PENG, TENG-based gas sensors work simply by the formation of a dipole layer after triboelectric contact and static separation between two materials of the triboelectric-series. The resistivity of the triboelectric material changes due to the chemisorptions of the molecular oxygen species by the surface, which can be affected by a bit of opposite charges [93, 94]. Since 2015, extensive works have been carried out to develop triboelectric-based self-powered active gas sensors, as elaborated in the following.

### ZnO-Based Triboelectric Materials

The working mechanism of TENG-based self-powered gas sensors can be ascribed to the coupling of triboelectrification effect and surface reaction of triboelectric materials. Among various triboelectric materials, although ZnO is not a tribo-series material, it exhibits triboelectric properties due to its finite conductivity characteristics. The discrete surfaces of ZnO NRs would be desirable for TENG, which require an efficient contact and interface to the surface of triboelectric polymer materials [95]. Regarding combination of outstanding gas sensing property and electrification ability, in 2015, Jeong Min Baik’s group proposed the first TENG-based self-powered gas sensor based on triboelectrification by the physical contact between the ZnO NWs and the dielectric layers polytetrafluoroethylene (PTFE) films, and the heterogeneous catalytic reaction occurring on the ZnO NWs and the decorated NiO nanoparticles (NPs).

**Fig. 8:**
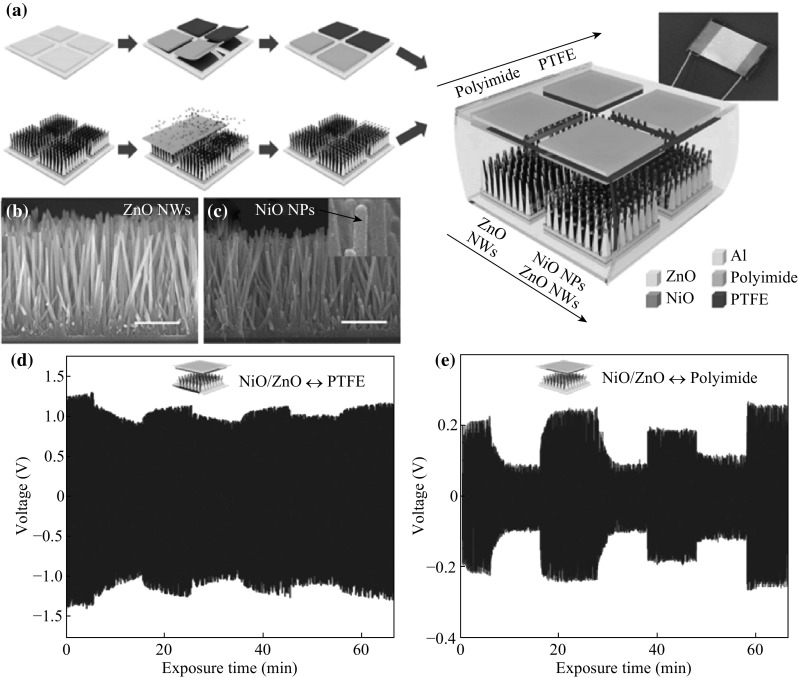
A self-powered, room-temperature electronic nose based on triboelectrification and heterogeneous catalytic reaction. **a** Schematic illustration of the fabrication process of a self-powered electronic nose and an optical image of the as-fabricated device. SEM images of **b** the ZnO NWs and **c** the NiO nanoparticle-decorated ZnO NWs grown on the SiO_2_/Si (*Scale bar*, 500 nm). The output performance of the sensor elements comprising **d** NiO nanoparticle-decorated ZnO-PFTE and **e** NiO nanoparticle-decorated ZnO-PI under cycled compressive force of 50 N at an applied frequency of 4 Hz. Figures adapted from [96]

The self-powered gas sensing system has been demonstrated in the form of electronic nose strategy with highly selective gas detection, as shown in Fig. 8. The electronic nose is a two-dimensional microarray where the individual sections orthogonally vary in their properties on account of two response-modifying strategies (Fig. 8a). Along one axis, a NiO NP functionality was applied to the ZnO NWs (Fig. 8b). The NiO functionality (Fig. 8c) was found to be more reactive for all volatile organic compound (VOC) gases, whereas only acetone gas was reactive on the surface of the ZnO NWs due to its small dissociation energy. The electron transfer to the NWs by the catalytic oxidation increased the triboelectric charge density at both surfaces, thereby increasing the output voltage of the devices. The slow response time also supported the contribution of the catalytic oxidation to the output power. Two dielectric layers [PTFE and polyimide (PI)] with different surface tensions were placed along the orthogonal axis. When the surfaces are exposed to the VOC gases, the output voltage decreases because the molecular species on both surfaces reduce the triboelectrically charged area. The sensor comprising a polyimide layer shows a faster response than the one including a PTFE layer, because of the higher surface energy of PI compared to that of PTFE (Fig. 10d, e). This self-powered sensor may be applicable in many places with limited accessibility to monitor gases and chemicals over long periods of time or in portable applications, such as electronic skins or textiles [96].

**Fig. 9:**
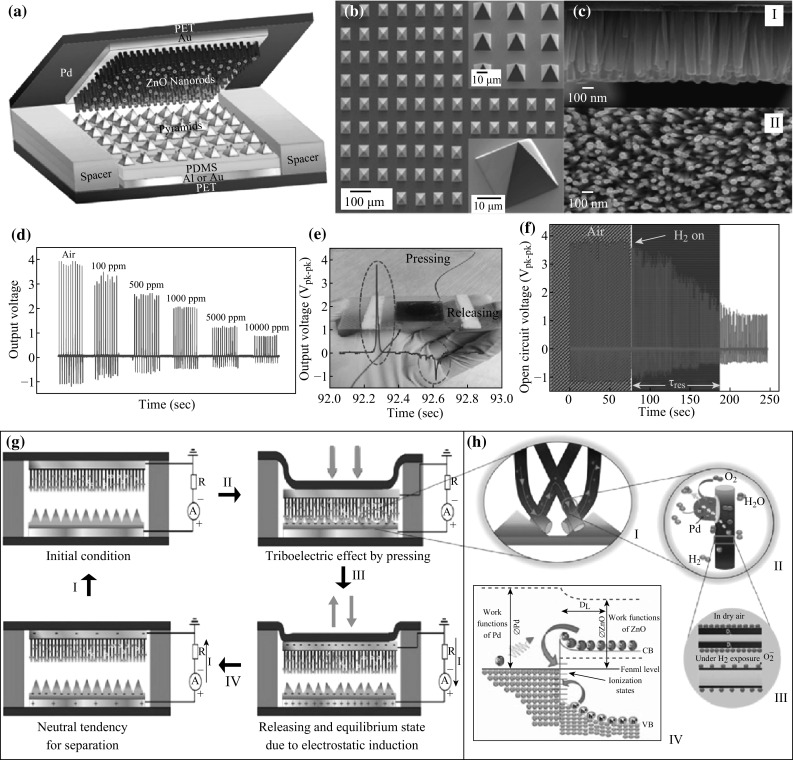
A self-powered active hydrogen gas sensor with fast response at room temperature based on triboelectric effect. **a** Schematic of the as-fabricated triboelectric-based H_2_ sensor (TEHS). **b** FESEM images of the micropatterned PDMS surface (the *insets* show the enlarged view of the micropyramids PDMS). **c** Cross-section (I) and in-plane (II) micrographs of ZnO nanoparticles. **d** Variation of open-circuit voltage with and without H_2_ exposure. **e** The pressing and releasing signal peaks of the TEHS. **f** Response time characteristics of the TEHS. Schematic of the TEHS’s **g** triboelectric and **h** sensing mechanisms. Figures adapted from [97]

In another work, A. S. M. Iftekhar Uddin and Gwiy-Sang Chung have successfully fabricated a triboelectric-based H_2_ sensor (TEHS) by combining a uniformly grown Pd NPs/ZnO NRs/Au/PET (polyester, PET) film with a micropyramid PDMS film. PDMS has already got considerable priority as a structure material due to its greater ability to attract and retain electrons upon contact with any positively charged triboelectric materials (Fig. 9a–c). In air, the TEHS device shows a peak-to-peak *V*_oc_ and *I*_sc_ of about 5.2 V and 80 nA, respectively. The as-fabricated device also shows an effective and reliable detection ability of H_2_ molecules at room temperature, in which a continuous decrease in output voltages with increasing H_2_ gas concentrations was observed (Fig. 9d). A maximum response of ∼373% and a short response time of 100 s were obtained, while the TEHS was exposed to 10,000 ppm H_2_ (Fig. 9e). The working mechanism of the TEHS device can be explained through the conjunction of the triboelectric effect and surface reaction mechanism. The polarized charges resulted from the friction (triboelectric effect) also regulated the charge carrier transport through the Pd/ZnO interfaces and modulated the interfacial energy at the junction area. Moreover, with the increasing H_2_ concentrations this phenomenon was accelerated, resulting in further decrease in output voltages (Fig. 9f, g). The aforementioned features of the TEHS device along with the major advantages in gas sensitivity, reliability, cost, scalability, durability and implementation will open up a new paradigm for widespread adoption of self-powered active H_2_ sensing in the near future.

### Non-ZnO-Based Triboelectric Materials

Except for ZnO nanomaterials, researchers have been looking for other good tribo- as well as gas sensing materials for self-powered gas sensing system, such as conductive polyaniline (PANI), which is a good gas sensing material since PANI chains can react with various volatile organic compounds at room temperature.

**Fig. 10:**
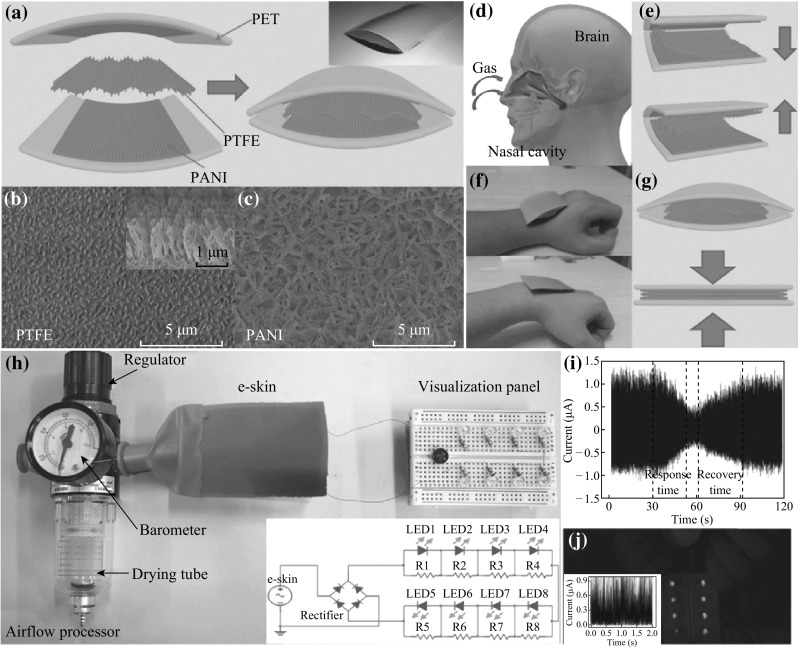
Outputting olfactory bionic electric impulse by PANI/PTFE/PANI sandwich nanostructures and their application as flexible, smelling electronic skin. **a** Structure and fabrication process of the flexible smelling e-skin (the *inset* shows the photograph of the e-skin). **b** SEM image of PTFE nanostructures on the *top view* (the *inset* shows cross-sectional SEM image of PTFE NWs). **c** SEM image of PANI nanostructures on the *top view*. **d** and **e** The e-skin can be easily driven by gas flow (human heavy breath or blowing). **f**, **g** The e-skin can be easily driven by body motion (pressure). **h** Photograph of the self-powered breath–alcohol visual detecting system. **i** Real-time continuous responding/recovering process of the e-skin against 210 ppm ethanol gas flow. **j** An adult without drinking alcohol can light 8 LEDs. Figures adapted from [98]

Xue et al. fabricated a flexible smelling e-skin that based on the triboelectrification/gas sensing coupling effect of PANI/PTFE/PANI sandwich nanostructures, as shown in Fig. 10. The PTFE film acts as the triboelectrification material. The two PANI films act as both the functional (sensing/triboelectrification) materials and the electrodes (Fig. 10a–c). The flexible e-skin has two operation modes: gas flow (human breath) driving the vibration of PTFE film (Fig. 10d, e); pressure (human motion) driving the movement of PANI films (Fig. 10f, g). The self-powered system consists of a gas flow processor, a smelling e-skin and a visualization panel. The visualization panel can directly display breath–alcohol concentration by counting the alight LED (Fig. 10h). The gas flow-induced output current/voltage is significantly dependent on the environmental atmosphere, which can act as olfactory bionic electric impulse. Against ethanol, the detection limit of the e-skin is 30 ppm, and the response is up to 66.8 against 210 ppm ethanol gas flow. Interestingly, the response of the e-skin keeps stable under different gas flow rates or with different device sizes/bending status. Also, the e-skin has relatively short response/recovery time (<25 s) and can detect various volatile organic compounds (Fig. 10i). When an adult without drinking alcohol blows the system, all the 8 LEDs can be lighted. When a drunken adult blows the system, the ethanol in the breath can dramatically decrease the output of the e-skin, and the alight LEDs are dependent on the ethanol concentration (Fig. 10j). Finally, an application of the flexible smelling e-skin for visually identifying drunken driver without any external electricity power has been demonstrated [98].

The present results shed light on designing new specialized-function e-skin and novel self-powered nanosystem. Some other non-ZnO materials have also been studied for TENG-based self-powered gas sensing system, such as Pd-functionalized ITO surface and PET film for self-powered H_2_ sensor [93], PDMS and PEDOT:PSS film with nylon fiber film for self-powered active acetylene gas sensing [99].

### Novel Mutual Independent System

The approach of realization of self-powered sensors normally is to actively generate electrical signal by itself as a response to the stimulation or triggering from the ambient environment, as described in above-mentioned works. However, it still requires external monitoring circuits to collect the signal generated by NGs, which means these sensing systems are not genuinely and authentically “self-powered” [19, 100, 101]. Obviously, developing appropriate NGs to directly charge or monitor sensors is another approach to achieve real “self-powered” system.

**Fig. 11:**
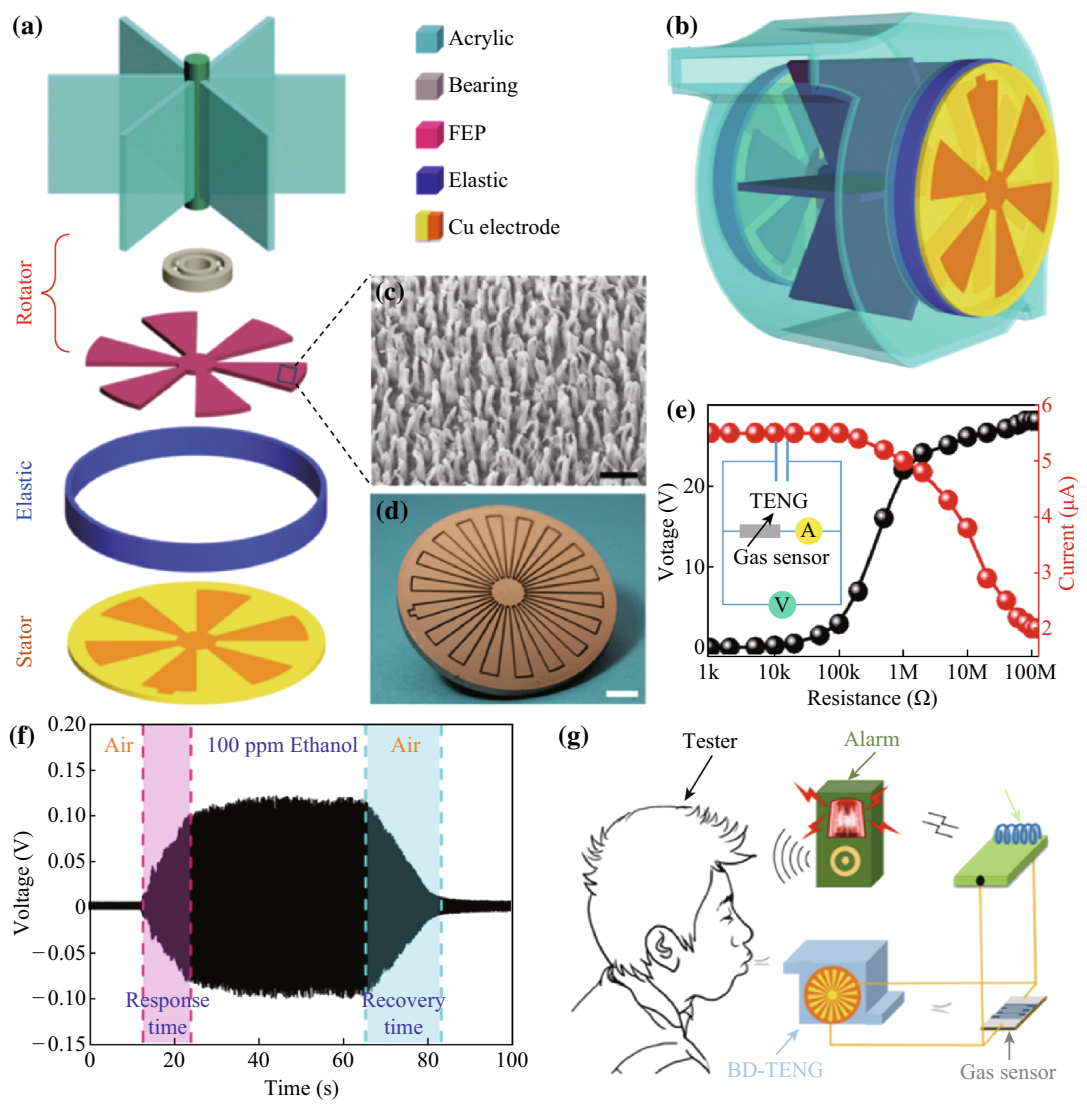
Blow-driven TENG as an active alcohol breathalyzer. **a** Schematic illustrations of the functional components of the blow-driven triboelectric nanogenerator, which mainly consists of three parts, a rotator, a stator and a soft elastic. **b** A SEM image of the FEP polymer NWs (*scale bar*, 500 nm). **c** A photograph of the as-fabricated stator (*scale bar*, 1 cm). **d** A schematic illustration of an as-fabricated device (*scale bar*, 2 cm). **e** The variation of the output voltage and current with external load resistances under a fixed blowing speed of 6 m/s (the *inset* shows the measurement circuit diagram). **f** A real-time continuously measured voltage profile to show the dynamic response to ambient alcohol concentrations. **g** A schematic illustration showing the self-power breathalyzer. Figures adapted from [102]

Facing this problem, Zhong Lin Wang’s group, for the first time, introduced a completely new working principle of self-powered gas sensing system by fabricating a blow-driven TENG to supply power for active alcohol detection, as shown in Fig. 11. The basic structure of the self-powered gas sensing system mainly consists of three functional parts, a traditional gas sensor, an alarm as well as a blow-driven TENG as power source, which can be driven by mouth blowing (Fig. 11a, b). The vertically aligned fluorinated ethylene propylene (FEP) NWs with the average clustering diameter of ~100 nm and length of ~1 μm have been prepared for enhancing triboelectric output (Fig. 11c). The stator, as one triboelectric material, is composed of two copper electrodes with complementary patterns, which have been separated by fine trenches in between (Fig. 11d). The alcohol vapor would dramatically increase the resistance of the sensor that led to an increased voltage drop across the sensor, when the blow-driven TENG was blown by a tester after alcohol drinking (Fig. 11e). The as-developed active alcohol breath analyzer based on the BD-TENG is featured as high detection response of about 34–100 ppm alcohol gas under an optimized sensor working temperature, fast response time of 11 s as well as a fast recovery of 20 s (Fig. 11f). A signal processing circuit diagram of a complete self-securing warning system which can be triggered by an increased voltage signal is shown in Fig. 11g.

The induced voltage across the sensor holds a proportional relationship with the breathed-out alcohol concentration regardless of the blow speed and quality airflow so that this blow-driven TENG-based self-powered gas sensor makes a significant progress toward the practical application. All the results indicate that the novel self-powered sensing system enables to work stably and reliably. Besides, given other competitive features, including being light weight, easy fabrication, cost-effectiveness, the justified concept in this work not only launches a new approach with extensive potential in the field of gas sensing, but also make a significant progress toward the practical application of a novel mutual independent TENG-based self-powered gas sensing system [102].

## Conclusions and Outlook

In this review, the establishment and latest progress in NG-based self-powered gas sensors are systematically summarized. We have also summarized recent researches about various NG-based self-powered gas sensors in Table 1. As an important functional application, self-powered gas sensors can operate independently based on two main technologies including PENG and TENG, without the use of external electricity storage/supply systems.

**Table 1:** A summary of various NG-based self-powered gas sensors

Type	Materials	Morphology	Gas	Concentration	Sensitivity	References
Piezo	ZnO	NW	H_2_S	100 ppm	13.1^A^	[59]
Piezo	ZnO	NS	Humidity	10 ppm	2.96^A^	[60]
Piezo	ZnO + UV	NW	Ethanol	700 ppm	85^A^	[103]
Piezo	Cu/ZnO	NW	H_2_S	1000 ppm	1045.76^B^	[66]
Piezo	Pd/ZnO	Nanoarray	Ethanol	800 ppm	108^B^	[67]
Piezo	Au/ZnO	NW Array	Ethanol	1200 ppm	72.1^A^	[68]
Piezo	Cd/ZnO	NW	Humidity	70% RH	85.7^A^	[70]
Piezo	Pt/ZnO	Nanoarray	Ethanol	1000 ppm	37.14^B^	[69]
Piezo	SnO_2_/ZnO	Nanoarray	H_2_	800 ppm	471.4^B^	[104]
Piezo	ZnSnO_3_/ZnO	NW	Liquefied petroleum	8000 ppm	83.23^A^	[105]
Piezo	*α*-Fe_2_O_3_/ZnO	NW Array	Ethanol	700 ppm	706.8^B^	[106]
Piezo	NiO/ZnO	NW	H_2_S	1000 ppm	84.3^B^	[107]
Piezo	In_2_O_3_/ZnO	Nanoarray	H_2_S	700 ppm	925^B^	[108]
Piezo	CuO/ZnO	Nanoarray	H_2_S	800 ppm	629.8^B^	[71]
Piezo	CeO_2_/ZnO	Nanoarray	Humidity	95% RH	82.1^A^	[109]
Piezo	*p*-Si/*n*-ZnO	NR	NO_2_	750 ppb(amine) 750 ppb(thiol)	23.5(amine)^A^ 12.8(thiol)^A^	[72]
Piezo	CdS	NR	H_2_S	600 ppm	62.5^A^	[73]
Tribo	NiO-ZnO PDMS	NW Film	Ethanol	0.1%	37.5^A^	[96]
Tribo	Pd/ZnO PDMS	NW Film	H_2_	10,000 ppm	373^B^	[97]
Tribo	PANI PTFE	Film Film	Ethanol	210 ppm	66.8^C^	[98]
Tribo	PEDOT:PSS Ag-ZnO/nylon	Film Fiber film	C_2_H_2_	1000 ppm	70.9 (indoor)^A^ 89 (outdoor)^A^	[99]
Tribo	Pd-ITO PET	Film film	H_2_	1%	75^A^	[93]
Tribo	Pd/ZnO PDMS	NW Film	H_2_	3 vol%	1457.69^B^	[94]
Tribo	Co_3_O_4_ Cu and PTFE	NR Film	Ethanol	100 ppm	34^D^	[102]

A: *S* % = (*V*_a_ − *V*_g_)/*V*_a_ × 100%; B: *S* % = (*V*_a_ − *V*_g_)/*V*_g_ × 100%; C: *S* % = (*I*_a_ − *I*_g_)/*I*_a_ × 100%; D: *S* = *V*_a_/*V*_g_, where *V*_a_ and *V*_g_ are the output voltages of the device under the same conditions in dry air and the test gas, respectively; *I*_a_ and *I*_g_ represent the output current in air and test gas, respectively

For PENG, it has been achieved in a series of works through the coupling of piezoelectric effect and gas sensing characteristics, in which the piezoelectric output of PENG acts as both the power source and sensing signal. The gas adsorption on the surface of piezoelectric-semiconductor materials can change the free-carrier density, which can vary the piezoelectric output upon applied deformation through the piezoelectric screening effect. However, compared with the traditional gas sensors, the conventional PENG-based self-powered gas sensors have some limitations. Firstly, the requirements of materials both having piezoelectric and semiconducting gas sensing properties, mainly referring to ZnO as building block, seriously restrict the most use of other non-piezoelectric but owning excellent gas sensing materials. Introducing noble metal decorated material, heterostructure and organic material-based gas sensors will further expand the scope for self-powered gas sensing systems. Secondly, due to their compact and completely sealed device structures, the response time of these gas sensors is in the range of tens of seconds, which is not fully well-suited for gas exposure on the sensing materials. Therefore, the complexities of the requirements demand to search for some simple sensor packaging system for better sensor performances. Thirdly, in most cases, the adsorption of gas molecules will happen for more than one kind, a better method of ascribing the variation of the piezoelectric output to a specific gas or optimizing its selectivity should be considered. Fourthly, theoretically, high piezoelectric potential would be generated when a large external force is applied onto a nanowire with a small thickness, because large deflection can be produced. However, in real cases, the deflection of nanodevice is restricted by their mechanical strength and flexibility.

While TENG-based self-powered gas sensors work simply by the formation of a dipole layer after triboelectric contact and static separation between two materials of the triboelectric series and the triboelectric charge density is greatly influenced by the surface alteration of certain chemisorptions of the molecular oxygen species. In comparison with PENG, even though most gas sensing materials are not in the tribo-series list, ZnO, SnO_2_, etc. exhibit triboelectric properties due to their finite conductivity characteristics, which are widening the range of choosing tribo-gas sensing materials. Meanwhile, a variety of structural designs also solve the problem of gas exposure on the sensing materials. However, it also exists some limitations, such as firstly, the introduction of motion will cause the mechanical disturbance of measuring equipment; Secondly, the output of TENG is unstable, and it may change with respect to the environment when used for real-time self-powered systems; thirdly, it still requires external monitoring circuits to collect the signal generated by NGs, which means these sensing systems are not genuinely and authentically “self-powered.” Developing appropriate TENGs to directly charge or monitor sensors is the trend of self-powered gas sensors. The whole system does not require external monitoring circuits to collect the signal, which have achieved genuinely and authentically “self-powered.” Meanwhile, the three main parts of this system work independently and do not interfere with each other. Moreover, the voltage output of TENG remains constant under various working frequencies. All the results indicate that the novel system enables to work stably and reliably, which provides a strong theoretical basis and technical support for the next generation of self-powered gas sensing system.

As a new field by coupling piezoelectric or triboelectric with semiconducting gas sensing characteristics, the NG-based self-powered gas sensing system has been demonstrated with sustainable, flexible, light weight, high efficient, cost-effective and environmental friendly designs. Continued progress in this field will lead to a class of self-powered gas sensors with superior sensitivity, excellent selectivity, high reliability and extended lifetimes for a wide range of environments and applications. It will be a collaborative developing field with various disciplines such as materials, energy, chemistry, automation, mechatronics and information.
